# Necrotizing Sarcoid Granulomatosis: A Disease Not to be Forgotten

**DOI:** 10.1155/2020/5730704

**Published:** 2020-01-27

**Authors:** A. I. Parejo-Morón, M. L. Tornero-Divieso, M. R. Férnandez-Díaz, L. Muñoz-Medina, O. Preda, N. Ortego-Centeno

**Affiliations:** ^1^Servicio de Medicina Interna, Hospital Universitario Clínico San Cecilio, Granada, Spain; ^2^UGC Enfermedades Infecciosas, Hospital Universitario Clínico San Cecilio, Granada, Spain; ^3^Servicio de Anatomía Patológica, Hospital Universitario Clínico San Cecilio, Granada, Spain; ^4^Unidad de Enfermedades Autoinmunes Sistémicas, Hospital Universitario Clínico San Cecilio, Granada, Spain; ^5^Instituto Biosanitario Granada, Granada, Spain; ^6^Departamento de Medicina, Universidad de Granada, Granada, Spain

## Abstract

Sarcoidosis is a systemic granulomatous disease of unknown aetiology characterised by the appearance of noncaseifying epithelioid granulomas in the affected organs, most commonly the lungs, skin, and eyes (Iannuzzi et al. 2007). Necrotizing Sarcoid Granulomatosis (NGS) is a rare and little-known form of disease, which also presents nodular lung lesions, and it shares pathologic and clinical findings with sarcoidosis, where the presence of necrosis may lead to misdiagnosis of tuberculosis (TB), leading to a consequent delay in treatment of the underlying entity (Chong et al. 2015). This is exactly what happened with the two cases that we present here.

## 1. Case Presentation

Case 1 was a 24-year-old woman who was 37 weeks pregnant, referred to our Internal Medicine unit because of a deep supraclavicular lymphadenopathy on the left side whose fine needle aspiration biopsy (FNAB) revealed granulomas with necrosis. Case 2 was a 31-year-old male with neurological symptoms (bradypsychia, peripheral vertigo, weakness in right lower limb, instability, and sphincter incontinence), whose cerebral nuclear magnetic resonance (NMR) revealed the presence of meningeal uptake; chest tomography scan (CT) showed mediastinal nodules and bilateral bronchoalveolar infiltrates; and the open lung biopsy showed sarcoid-like granulomas with extensive necrosis. Both patients received initially standard antituberculous treatment, but due to lack of response, the possibility of a necrotizing sarcoid granulomatosis raised up. After the start of treatment with glucocorticoids, the evolution was favourable in both cases.  Table 1 provides more details of these cases.

## 2. Discussion

Necrotizing sarcoid granulomatosis was first described in 1973 by Liebow, who noted the histological presence of confluent epithelioid granulomas with small central necrosis foci or more extensive necrosis, as well as vasculitis [1]. Liebow diferentiated this granulomatous disease from other forms of noninfectious pulmonary angiitis and granulomatosis: Wegener's granulomatosis, Churg–Strauss syndrome, bronchocentric granulomatosis, and lymphomatoid granulomatosis. Actually most authors consider the entity as a form of sarcoidosis more than a distinct entity, differing in the fact that there is more intense necrosis and vasculitis [2].

Clinically, very few differences have been described between the two variants: classical and necrotizing, with pulmonary involvement predominating in both.  Table 2 provides more detail on differences between them. In case 1, the clinical manifestation was a supraclavicular lymphadenopathy; peripheral lymphadenopathy appears in 40% of sarcoidosis patients. It should be noted that the presence of intrathoracic lymphadenopathies is more frequent in the classic form (85%) than in the necrotizing form (33%). In case 2, the predominant manifestation was central nervous system (CNS) involvement, which appears in 5.78% of NGS patients [3] and in the same proportion in patients with the classic form [4].

In respect of tuberculosis, the most frequent clinical presentation is also pulmonary involvement. The most frequent extrapulmonary form is lymph node tuberculosis, which is responsible for 43% of peripheral lymphadenopathies in the developed world [6]; CNS involvement is rarer (5.5%); due to this, it requires haematogenous dissemination either from a distal focus or during disseminated TB [7].

Correct diagnosis is vital because of the different treatments for the pathologies. Necrotizing sarcoidosis has a good response to corticoids, becoming benign [2, 5], and exceptionally severe neural involvement leading to death has been reported [3]. In contrast, tuberculosis disease requires TB treatment over a period of time that depends on the area affected [7].

Given the low effectiveness of cultures from most extrapulmonary locations for studying extrapulmonary tuberculosis, biopsy may be required for diagnosis. Visualising granulomas with caseification necrosis in biopsy samples, together with a compatible medical history, is practically diagnostic. Even so, samples should always be processed for microbiological study (staining, PCR, and culturing). A lack of microbiological isolation in the samples should therefore lead us to suspect a different granulomatous disease, such as necrotizing sarcoid granulomatosis [8].

Similarly, in patients suspected of having an active tuberculosis infection (based on clinical radiological findings), it is recommended to initiate the antituberculous regimen prior to microbiological isolation and evaluate the response after 2-3 weeks, while the microbiological results are being prepared. If there are no clinical or radiological changes and the microbiological study is negative, steroid therapy can be started [8].

In the two cases presented here, TB treatment was initiated. Given the absence of a favourable response, it was performed a biopsy that led to the diagnosis, allowing corticoid treatment to be initiated. In both patients, good disease control was achieved with low doses of corticoids, combined with methotrexate in case 2, permitting rapid reduction in prednisone doses.

## 3. Conclusion

Necrotizing sarcoid granulomatosis should be considered within the differential diagnosis of granulomatous diseases (Table 3), and knowledge of this variant is essential in order not to rule out sarcoidosis due to the presence of necrosis. The extended duration of the disease, its glucocorticoid response, negative cultures, and lack of response to TB treatment make it less likely for the aetiology to be infectious [8].

## Figures and Tables

**Figure 1:**
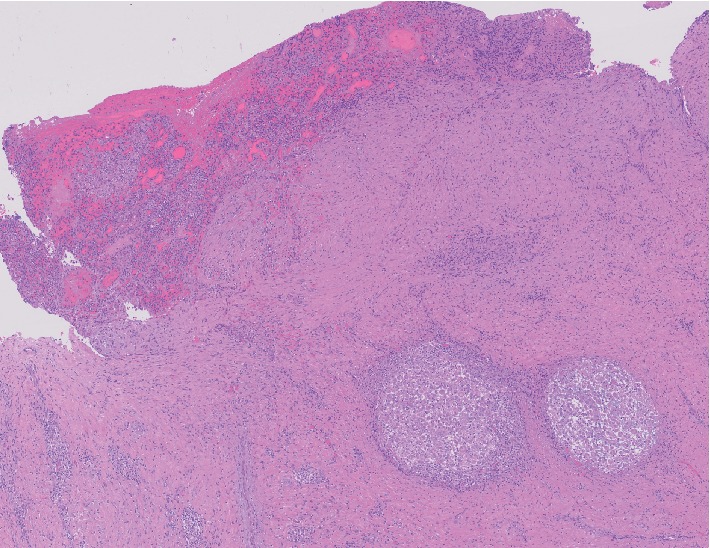
Histopathology of the supraclavicular ganglion exeresis (case 1). Nodular granulomatosis with extensive necrosis replacing the normal tissue architecture (haematoxilina-eosina, magnified ×4).

**Table 1:** Clinical characteristic of the two patients with necrotizing sarcoid granulomatosis. SACE: serum angiotensin-converting enzyme; ACE: angiotensin-converting enzyme; ADA: adenosine deaminase; PCR: polymerase chain reaction; BAL: bronchoalveolar lavage; AFB: acid-fastness; CT: tomography scan; EEG: electroencephalography; NMR: nuclear magnetic resonance; and FNAB: fine needle aspiration biopsy.

	Case 1	Case 2
Sex and age	24-year-old female. 37 weeks pregnant	31-year-old male
Family history	Father diagnosed with discoid lupus	Brother diagnosed with sarcoidosis (pulmonary and cutaneous involvement)

Presentation	Left supraclavicular lymphadenopathy	Peripheral vertigo, weakness in right lower limb, instability, and sphincter incontinence

Physical examination	Approx. 4 × 4 cm supraclavicular tumour attached to deep planes	Bradipsychia. Right horizontal nystagmus. Paresis 4+/5 left upper limb and lower limbs. Left extensor cutaneous plantar reflex. Unstable romberg

Laboratory	No lymphopoenia. T CD4/CD8 lymphocyte ratio: 1.43. Normal SACE. Calcium/phosphorus metabolism: normal. 24 h urine calciuria slightly higher than normal (264 mg/dL). Positive Mantoux.	Discrete lymphopoenia. T CD4/CD8 lymphocyte ratio: 0.97. High SACE. Calcium/phosphorus metabolism: normal. Calciuria in urine at 24 h: normal. Positive Mantoux. Lumbar puncture: High ACE and ADA. Cultures (including fungi) and indian ink: negative. Sputum culture and mycobacterial PCR: negative. BAL and sputum samples: negative for AFB

Imaging tests	Cervical CT scan, lymphadenitis that does not suggest pyogenic origin. Chest x-ray: normal.	EEG: delta activity, more frequent on the right side. Brain NMR: meningeal uptake that extends to the cervical area. Chest CT scan: mediastinal nodules and bronchoalveolar infiltrates in both bases

Anatomical pathology	FNAB supraclavicular adenopathy: necrosis and granulomas. PCR mycobacterium tuberculosis: negative. Ganglion exeresis: chronic lymphadenitis with sarcoid granulomas (Figure 1)	Open lung biopsy: necrotizing granulomatous infiltrates. PCR mycobacterium tuberculosis: negative

**Table 2:** Characteristics of classical variant (nodular sarcoidosis) and necrotizing variant (NGS) [2, 5].

	Nodular sarcoidosis	Necrotizing variant
Epidemiology	Prevalence: 10 to 20 per 100,000 population Males 44% Females 56% Median age: 35	<300 cases have been reported Males 37% Females 63% Median age: 42
Histology	Nonnecrotizing epithelioid granulomas	Granulomas Necrosis (coagulative or caseous) and vasculitis Foci of infarction
Clinical presentation	88% Pulmonary and/or systemic symptoms (fever, weight loss, night sweats, malaise, and so on)	84% Pulmonary and/or systemic symptons (fever, weight loss, night sweats, malaise, and so on)
Involved organs		
>>SACE elevation	17%	4%
>>Eye involvement	14%	12%
>>Skin involvement	10%	2%
>>Lymphadenopathy	9%	0.5%
>>Liver involvement	9%	1%
>>Erithema nodosum	3%	1%
>>Sjögren or sicca syndrome	1%	3%
>>CNS involvement	2%	7%
>>Neuropathy	0%	2%
>>Splenic involvement	2%	1%
>>Lacrimal gland involvement	1%	2%
Diagnosis	Transbronchial lung biopsy (35%) Tissue obtained by surgical procedures (33%) Needle biopsy (9%) Bronchial biopsy (2%) Intrathoracic lymph node biopsy (8%) Extrathoracic lymph node biopsy (3%)	Tissue obtained by surgical procedures (98%)

SACE: serum angiotensin converting enzyme; CNS: central nervous system.

**Table 3:** Main differential diagnosis of NGS and their typical characteristics. GPA: granulomatosis with polyangiitis; FSGS: focal segmental glomerulosclerosis; EGPA: eosinophilic granulomatosis with polyangiitis; TB: tuberculosis; NTM: nontuberculous mycobacteria; BAL: bronchoalveolar lavage; ANCA: antineutrophil cytoplasmic antibodies; CRP: C-reactive protein; SIADH: syndrome of inappropriate antidiuretic hormone secretion; TC: computed tomography; and AFB: acid-fast bacilli.

	GPA	EGPA	TB	NTM
Epydemiology	Mean age at diagnosis: 40–60 years No gender predominance	Mean age at diagnosis: 40 years No gender predominance	100 per 100,000 or higher: Sub-Saharan Africa, India, and the islands of Southeast Asia and Micronesia 26 to 100 cases per 100,000: China, central and South America, Eastern Europe, and northern Africa Less than 25 cases per 100,000: United States, Western Europe, Canada, Japan, and Australia	Environmental contaminants in soil and water, having been isolated from the domestic water distribution network, hot tubs, swimming pools, and workplaces
Histology	Granulomatous inflammation, vasculitis, and necrosis	Eosinophilic infiltration Areas of necrosis Interstitial and perivascular necrotizing granulomas An eosinophilic, giant cell vasculitis, especially of the small arteries and veins	Granulomas caseating which contain epithelioid macrophages, Langhans giant cells, and lymphocytes	Granulomatous inflammation
Clinical presentation	Constitutional symptoms (fever, malaise, anorexia, and weight loss) Ear, nose, and throat manifestations (nasal crusting, sinusitis, otitis media, earache, otorrhea, persistent rhinorrhea, purulent/bloody nasal discharge, oral and/or nasal ulcers, and polychondritis) Tracheal and pulmonary disease (nodules cavitary, and pulmonary opacities) Renal manifestations (FSGS)	Poorly controlled asthma and lung disease (migratory infiltrates, pleural effusion, nodules rarely cavitary, and alveolar hemorrhage) Upper airway and ear disease Skin involvement Peripheral neuropathy (mononeuritis multiplex)	Constitutional symptoms (fever, malaise, and weight loss) Primary disease: pleuritic chest pain, fatigue, cough, arthralgia, and pharyngitis	Persistent fever, night sweats, weight loss, fatigue, malaise, and anorexia Pulmonary disease Superficial lymphadenitis Skin and soft tissue infection
Laboratory tests	ANCA positive Urinary sediment disorder	Peripheral blood eosinophilia ANCA positive BAL: high percentage of eosinophils in the lavage fluid	CRP elevated. Leukocytosis Hyponatremia, may be associated with the SIADH	Elevated acute phase reactants
Diagnosis	Biopsy of a site of suspected active disease	Surgical lung biopsy	Radiographic imaging (radiography and TC) and microbiologic testing (sputum AFB smear, mycobacterial culture, and molecular tests)	Recurrent isolation of mycobacteria from sputum or isolated from at least one bronchial wash in a symptomatic patient Culture of blood for mycobacteria
